# Supplementary material for: Interim safety and efficacy of gene therapy for RLBP1-associated retinal dystrophy: a phase 1/2 trial
Source: Nat Commun. 2024 Sep 10;15:7438. doi: 10.1038/s41467-024-51575-4 (PMC11387776; doi:10.1038/s41467-024-51575-4)
Supplement: Supplementary file 1 — Supplementary Information [file 41467_2024_51575_MOESM1_ESM.docx]

**Supplementary Information**

**Supplementary figures and tables**

**Supplementary Figure 1. AAV8-*RLBP1* construct.** AAV8-*RLBP1*, is a replication-deficient recombinant adeno-associated virus-based vector (rAAV) composed of an rAAV serotype 8 capsid and a self-complementary serotype 2 genome. The vector genome carries a gene expression cassette that expresses the human *RLBP1* gene under the transcriptional control of an endogenous human *RLBP1* promoter. This expression cassette includes a human *RLBP1* promoter sequence (nucleotides -3157 to -2568, including part of exon 1), a simian virus 40 (SV40) intron, the cDNA encoding human *RLBP1* and a SV40 polyadenylation signal sequence.

**Supplementary Figure 2. Subretinal bleb positions.** Color fundus images of subretinal bleb positions (red line) for each patient. The retinotomy site is marked (X). Blebs predominantly formed centrally (C1.A, C1.B, C1.C, C3.A, C3.B, C4.A and C4.C) or peripherally (C2.A, C2.B, C2.C, C3.C and C4.B).

**B**

**A**

**Supplementary Figure 3. Visual acuity outcomes.** (A) Best corrected visual acuity (BCVA) and (B) low-luminance visual acuity (LLVA) LogMAR-converted values for each patient at all available visits. Acuity values dropped for all patients at postoperative day 2 (D2) due to the remaining air tamponade. The BCVA returned to pre-treatment values by day 15 in all patients except for patient C4.C. Source data are provided as a Source Data file.


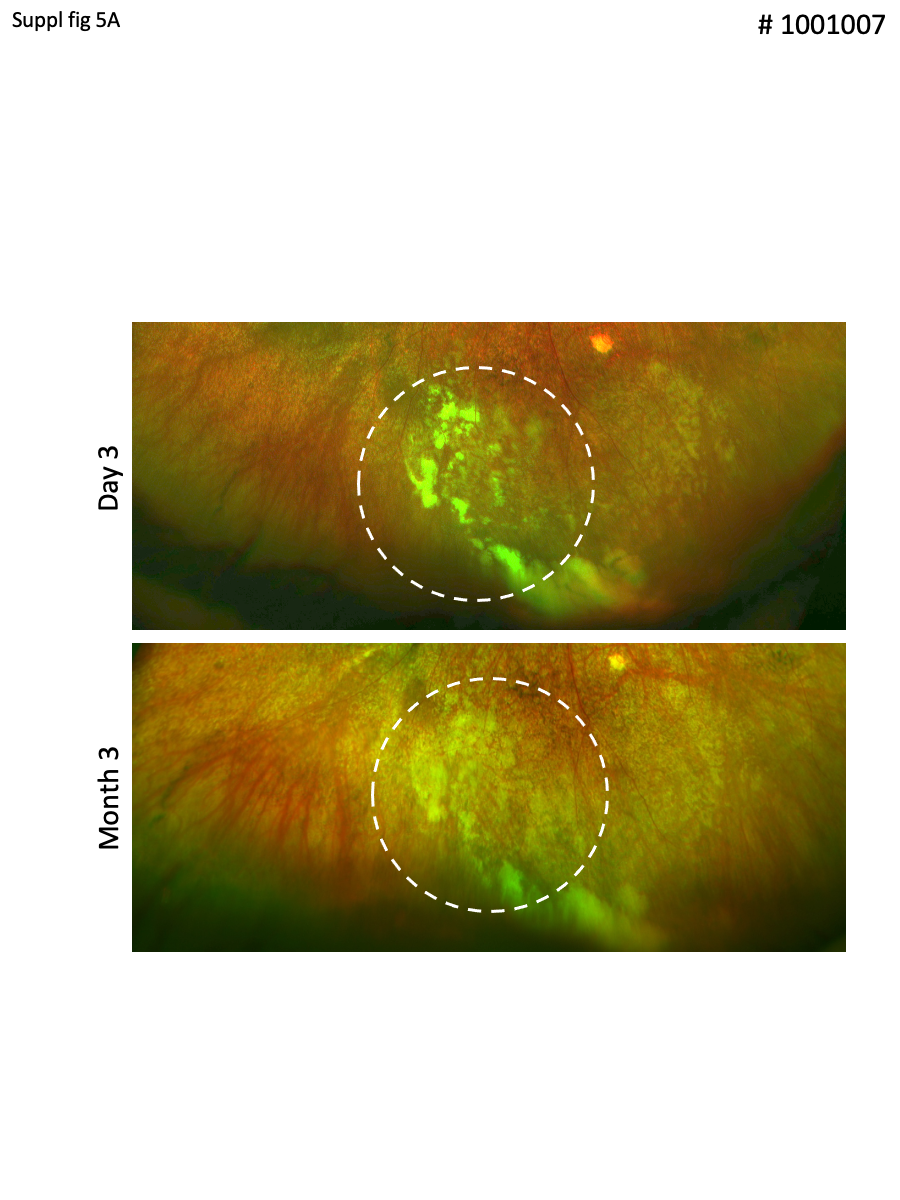


**B**

**A**


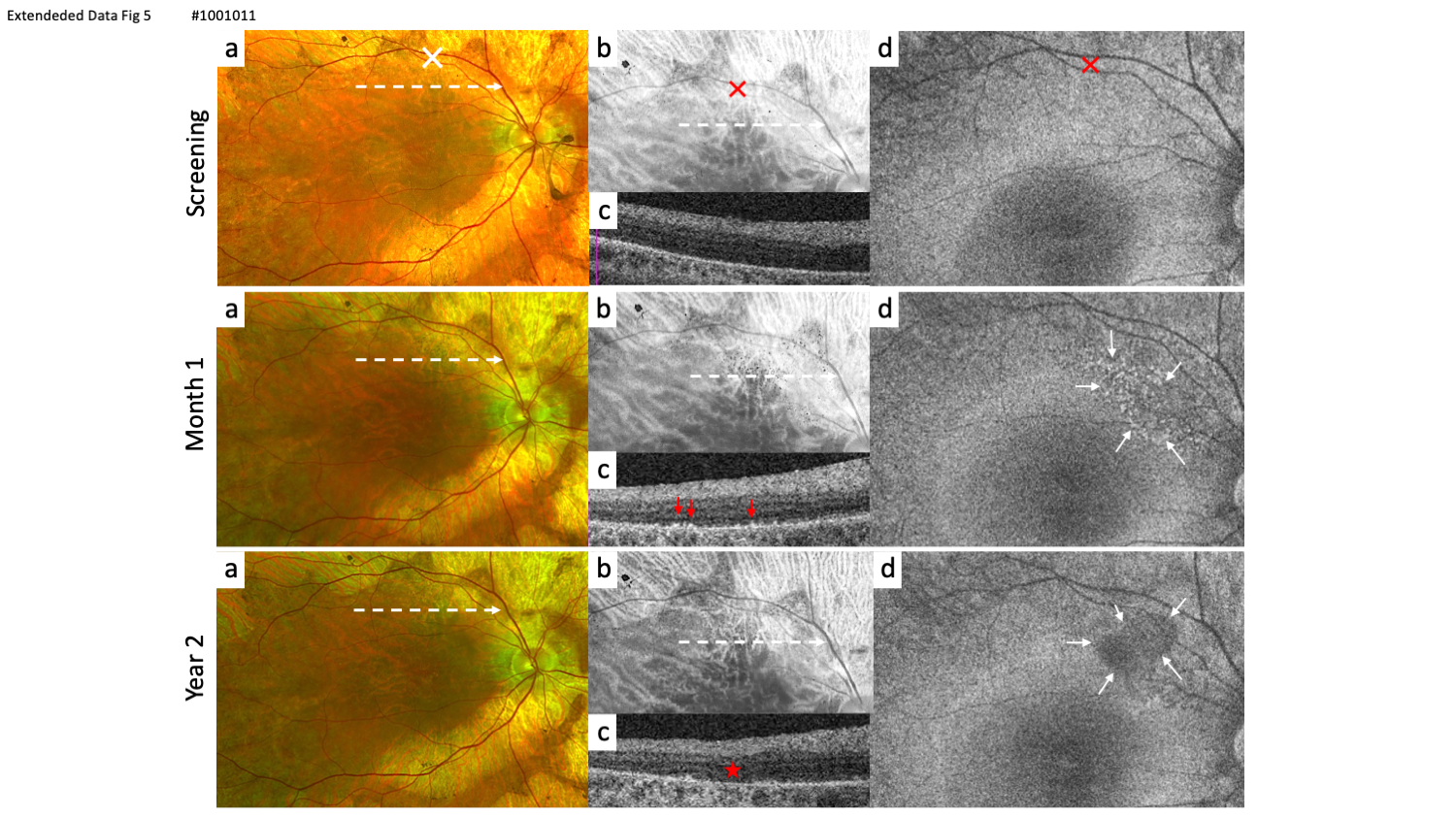


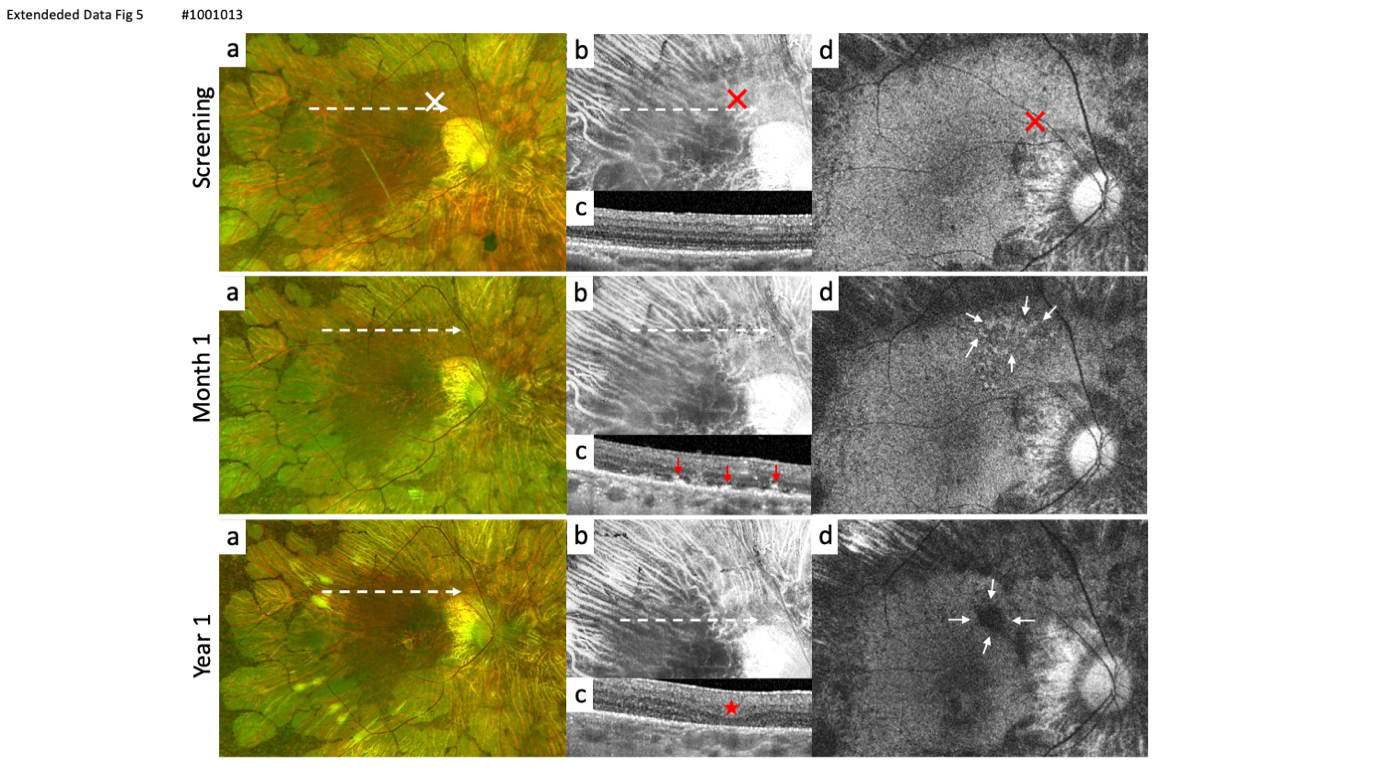


**C**

**Supplementary Figure 4.** **Examples of inferior subretinal exudates and subretinal pigmented deposits.** (A) Inferior subretinal exudates (dashed area) in patient C2.C three days after treatment with AAV8-*RLBP1*. Three months post-treatment the exudates had largely regressed. Subretinal pigmented deposits and secondary RPE atrophy in patient C4.A (B) and C4.C (C). Color fundus (a), enhanced red channel (b), SD-OCT scan (c) and FAF images (d) at screening, 1 month and latest post-treatment visit. Pigmented deposits present as hyperreflective dots at the RPE level (red arrows) corresponding to hyperfluorescent dots (area outlined by white arrows) on FAF. Secondary atrophy present as a hypofluorescent area of patchy loss of RPE (red star). SD-OCT scan planes (dashed arrow) and the retinotomy site of the subretinal bleb (X) are shown.


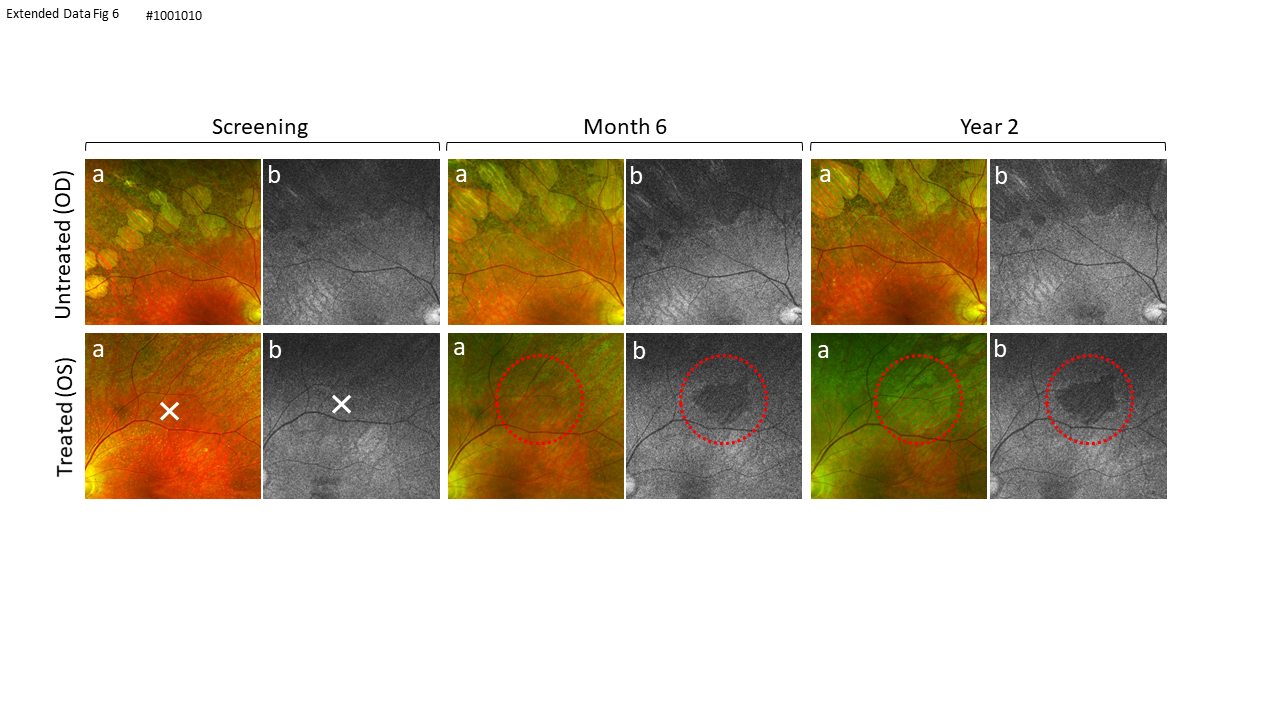

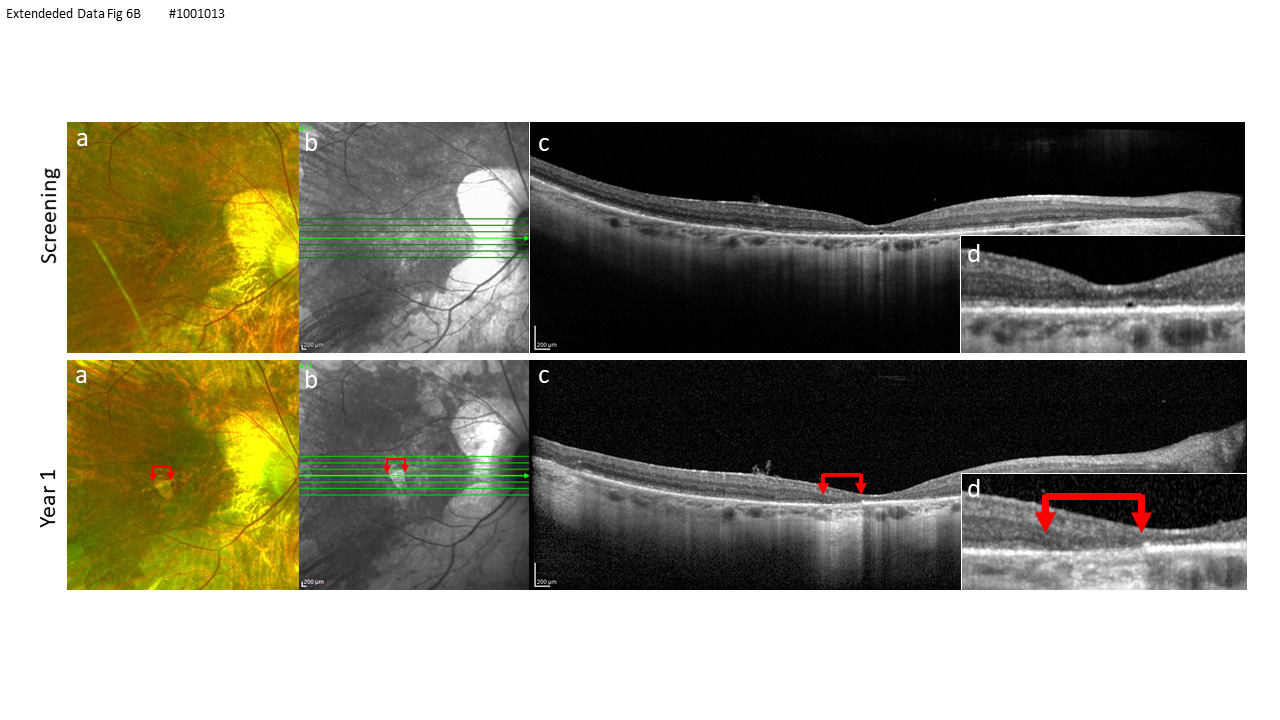


**B**

**A**

**Supplementary Figure 5. Examples of retinal atrophy.** (A) Color fundus (a) and FAF (b) images of RPE atrophy (dotted circle) at the retinotomy site of the subretinal injection is shown for patient C3.C. The retinotomy site is indicated (X). (B) Foveal atrophy in patient C4.C 1-year post-treatment is shown in the color fundus image (a). SD-OCT (c) scan through the juxtafoveal atrophy (green arrow in (b)) shows focal loss of the RPE and photoreceptor outer layers (between red arrows in (c) and (d)). Inserts show magnification of the foveal region (d).

**Supplementary Figure 6. Spectral-domain optical coherence tomography (SD-OCT) outcomes.** Change in SD-OCT-assessed retinal thickness for all 9 subfield sectors for all patients and available visits. The predefined 40% safety threshold is shown (dashed grey line). Colored lines are defined in the inserted box at the bottom left and refer to screening visit 1 (Scr1), screening visit 2 (Scr2), baseline visit (Bas), day 15 (D15), month 1 visit (M1), month 3 visit (M3), month 6 visit (M6), month 9 visit (M9), year 1 visit (Y1), year 2 visit (Y2) and year 3 visit (Y3). Source data are provided as a Source Data file.

**A**

**B**

**C**

**Supplementary Figure 7. Analysis of full-field dark adaptation kinetics.** (A) Dark adaptation curve of a healthy volunteer and a patient with *RLBP1*-RD pre-treatment. The patient’s pre-treatment dark adaptation curve shows the average of 3 pre-treatment values with the prediction interval. (B) Dark adaptation curves pre- and post-treatment of patient C1.A (C). A sample dot plot for a patient for a given time post-bleach for three post-treatment visits. Details on the assessment of the dark adaptation kinetics method are outlined in the Methods.

**Supplementary Figure 8. Dominant eye test outcomes.** Individual patient level data of dominant eye test outcomes at all available visits.

**A**

**B**

**C**

**Supplementary Figure 9. Patient reported outcomes (PROs).** Mean scores for PROs for the VFQ-25 (A) and LLQ (B) visual function questionnaires and individual data from all patients for a subset of PRO subscales (C) at available visits. Outcomes for patient C2.C that was treated in the dominant eye are highlighted (bold line). Error bars show standard deviation (SD). (A) Visit (n), Scr (12), M3 (12), M6 (12), M9 (6), Y1 (12), Y2 (10), Y3 (11). (B) Visit (n), Scr (12), M3 (8), M6, (12), M9 (6), Y1 (6), Y2, (6), Y3 (4). Source data are provided as a Source Data file.

**A**

**B**

**Supplementary Figure 10. Visual field outcomes.** Light-adapted microperimetry (A). The area under the cumulative distribution curve (AUC) for retinal sensitivity is shown for the treated and untreated eye of each patient at all available visits. Humphrey visual field mean deviation (B) is shown for the treated and untreated eye of each patient at all available visits. Missing 2-year data for patients C1.A and C1.B due to Covid-19. Source data are provided as a Source Data file.


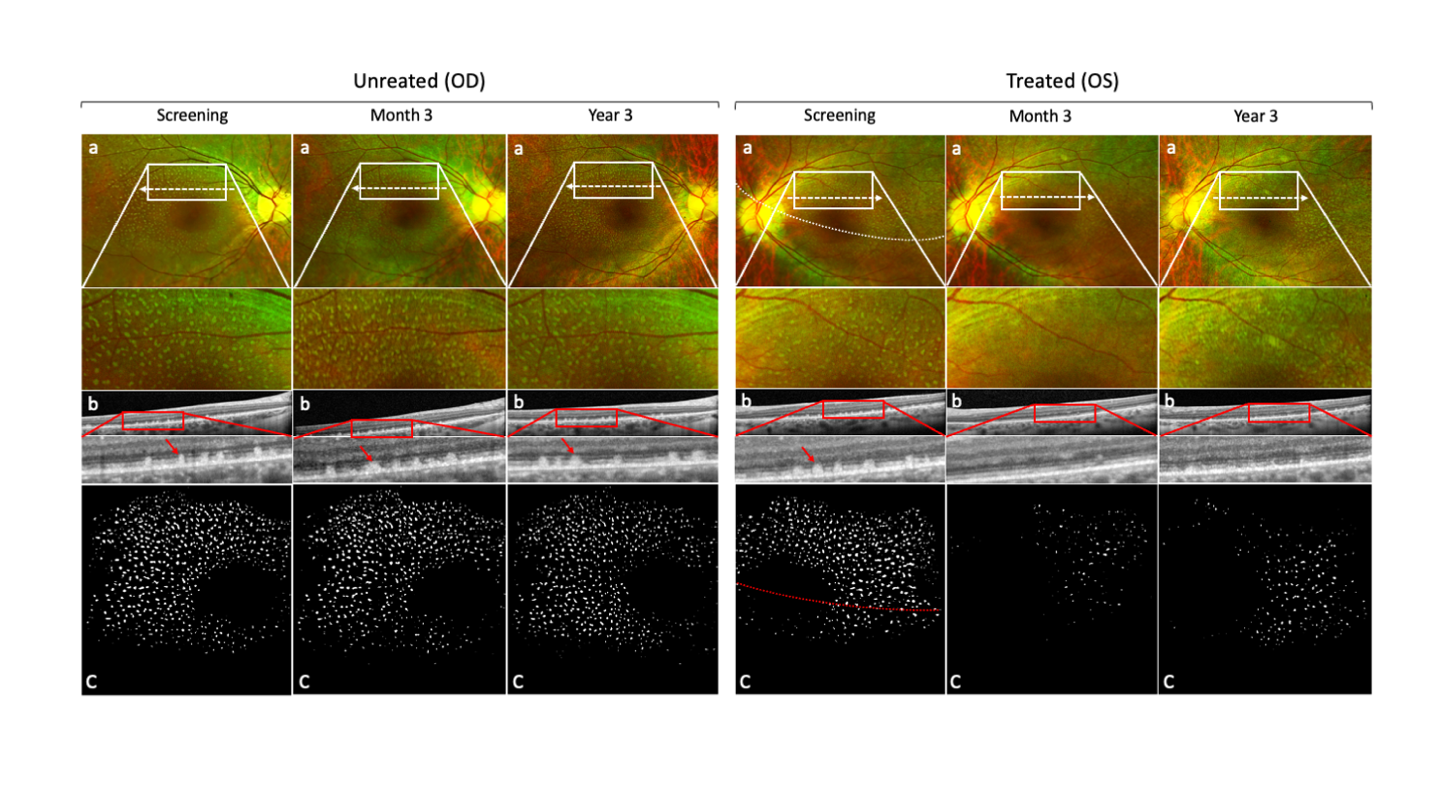


**A**


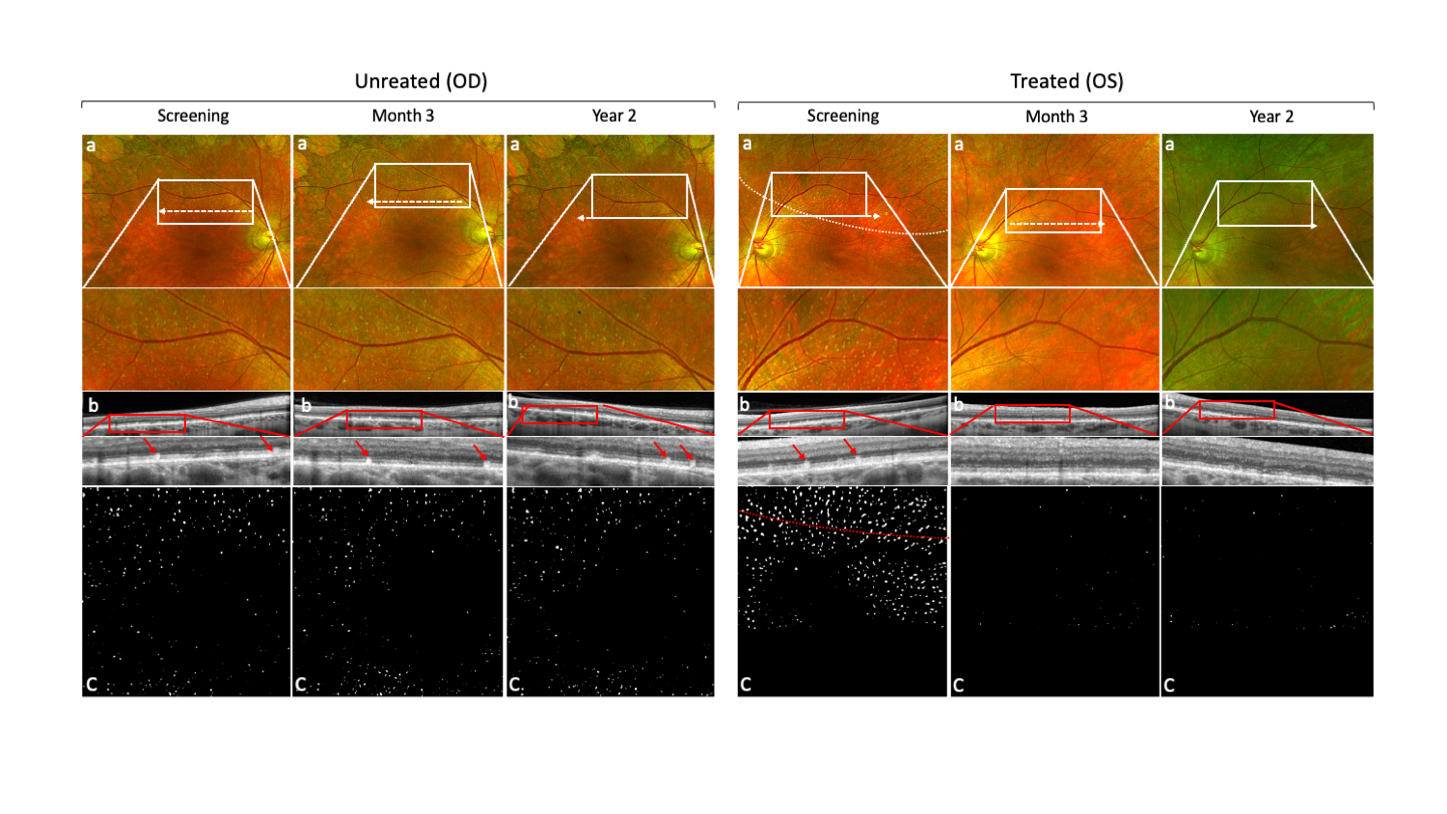


**C**

**B**

**Supplementary Figure 11. Regression and analysis of subretinal white puncta.** Punctata albescens deposits in patient C2.B (A) and C3.C (B) at screening, month 3 and latest available visit. Color fundus photos (a) and corresponding SD-OCT scan (b) show the puncta at the RPE level (red arrows). SD-OCT scan planes (dashed arrows in (a)) and the subretinal bleb border (dotted lines in (a) and (c)) are shown. Processed threshold *en face* images (c) are shown for the treated and untreated eye. (C) Example of image processing and thresholding for quantification of number of punctata albescens deposits further described in Methods. Original image (Left), image after processing (Middle) and image after processing and thresholding (Right).

**Supplementary Table 1. Adverse events (AEs).** Total incidence of AEs per treatment cohort. Source data are provided as a Source Data file.

^^

**Supplementary Table 2. Subjective functional narratives.** Subjective functional narratives were captured from the latest visit for each participant. The patients were asked if they observed any difference (better, same or worse) in night vision comparing treated eye to untreated eye. The patients were also asked to give specific daily-life examples of changes in visual function. ^1^3-years follow-up. ^2^2-years follow-up. ^3^1-year follow-up.
