# Supplementary material for: Interim safety and efficacy of gene therapy for RLBP1-associated retinal dystrophy: a phase 1/2 trial
Source: Nat Commun. 2024 Sep 10;15:7438. doi: 10.1038/s41467-024-51575-4 (PMC11387776; doi:10.1038/s41467-024-51575-4)
Supplement: Supplementary file 3 — Reporting Summary [file 41467_2024_51575_MOESM3_ESM.pdf]

nature portfolio

Corresponding author(s): Anders Kvanta
Last updated by author(s): Jun 18, 2024

Reporting Summary

Nature Portfolio wishes to improve the reproducibility of the work that we publish. This form provides structure for consistency and transparency in reporting. For further information on Nature Portfolio policies, see our Editorial Policies and the Editorial Policy Checklist.

Statistics

For all statistical analyses, confirm that the following items are present in the figure legend, table legend, main text, or Methods section.

n/a	Confirmed	
☐	☒	The exact sample size (*n*) for each experimental group/condition, given as a discrete number and unit of measurement
☒	☐	A statement on whether measurements were taken from distinct samples or whether the same sample was measured repeatedly
☐	☒	The statistical test(s) used AND whether they are one- or two-sided<br>*Only common tests should be described solely by name; describe more complex techniques in the Methods section.*
☒	☐	A description of all covariates tested
☒	☐	A description of any assumptions or corrections, such as tests of normality and adjustment for multiple comparisons
☐	☒	A full description of the statistical parameters including central tendency (e.g. means) or other basic estimates (e.g. regression coefficient) AND variation (e.g. standard deviation) or associated estimates of uncertainty (e.g. confidence intervals)
☐	☒	For null hypothesis testing, the test statistic (e.g. *F*, *t*, *r*) with confidence intervals, effect sizes, degrees of freedom and *P* value noted<br>*Give P values as exact values whenever suitable.*
☒	☐	For Bayesian analysis, information on the choice of priors and Markov chain Monte Carlo settings
☒	☐	For hierarchical and complex designs, identification of the appropriate level for tests and full reporting of outcomes
☒	☐	Estimates of effect sizes (e.g. Cohen's *d*, Pearson's *r*), indicating how they were calculated

Our web collection on statistics for biologists contains articles on many of the points above.

Software and code

Policy information about availability of computer code

Data collection	Diagnosys Colordome Software Version V6.64.15, Heidelberg Eye Explorer version 1.10.4.0, Optos Advance (ver 4.4R2), Nidek MP-3 (ver 1.1.2.00), Humphrey (ver 5.1.2)
Data analysis	Heidelberg Eye Explorer version 1.10.4.0, GraphPad Prism 9, Microsoft PowerPoint, ImageJ 1.54b, SAS version 9.4

For manuscripts utilizing custom algorithms or software that are central to the research but not yet described in published literature, software must be made available to editors and reviewers. We strongly encourage code deposition in a community repository (e.g. GitHub). See the Nature Portfolio guidelines for submitting code & software for further information.

Data

Policy information about availability of data

All manuscripts must include a data availability statement. This statement should provide the following information, where applicable:

- Accession codes, unique identifiers, or web links for publicly available datasets
- A description of any restrictions on data availability
- For clinical datasets or third party data, please ensure that the statement adheres to our policy

Due to the small sample size and patient privacy restrictions, it is unlikely that individual patient data can be shared. All requests for raw and analyzed data and materials are promptly reviewed by the St. Erik Eye hospital and the sponsor, Novartis Pharma AG, to verify whether the request is subject to any intellectual

property or confidentiality obligations or patient privacy issues. Patient-related data not included in the paper were generated as part of the clinical trials and may be subject to patient confidentiality. Any data and materials that can be shared will be released via a material transfer agreement upon reasonable request.

Research involving human participants, their data, or biological material

Policy information about studies with human participants or human data. See also policy information about sex, gender (identity/presentation), and sexual orientation and race, ethnicity and racism.

Reporting on sex and gender	This is an ultrarare disease for which there was no possibility to recruit subjects based on sex/gender distribution. We report only aggregated numbers of sex distribution in order to limit indirect patient identification.
Reporting on race, ethnicity, or other socially relevant groupings	This is an ultrarare disease for which there was no possibility to recruit subjects based on race, ethnicity or social grouping. We report only aggregated numbers of race in order to limit indirect patient identification.
Population characteristics	The trial population consisted of 12 participants with Bothnia-type RLBP-1 associated retinal dystrophy and genetically confirmed biallelic mutations in the RLBP1 gene.
Recruitment	The trial was a single center first-in-human Phase 1/2 interventional, non-randomized, non-confirmatory, open-label single ascending dose, gene replacement-therapy study to assess the safety, tolerability, and efficacy of AAV8-RLBP1 vector in patients with biallelic loss-of-function mutations in the RLBP1 gene. The study site was St. Erik Eye hospital, Stockholm. Sweden. Clinicaltrials.gov ref: NCT03374657, "CPK850" trial. Trial participants were recruited if they were willing and able to provide written consent and in the trial could fulfill the incusion and exclusion criteria as described in the Methods section of the manuscript.
Ethics oversight	The trial was performed with approval from the Swedish National Ethics Committee (Etikprövningsmyndigheten, EPM) and the Swedish Medical Products Agency (Läkemedelsverkat, LV) and adhered with all applicable laws and regulations including the International Conference on Harmonization Guidelines of Good Clinical Practice and the Declaration of Helsinki. Written informed consent was obtained from each participant.

Note that full information on the approval of the study protocol must also be provided in the manuscript.

Field-specific reporting

Please select the one below that is the best fit for your research. If you are not sure, read the appropriate sections before making your selection.

☒ Life sciences ☐ Behavioural & social sciences ☐ Ecological, evolutionary & environmental sciences

For a reference copy of the document with all sections, see nature.com/documents/nr-reporting-summary-flat.pdf

Life sciences study design

All studies must disclose on these points even when the disclosure is negative.

Sample size	The trial included 12 participants in 4 dose-escalation cohorts. A patient was considered a responder if there was improvement in dark adaptation recovery at a minimum of 2 or more consecutive post-treatment visits within the 1-year follow-up. Statistical analysis was conducted at the cohort level (based on responder rates at a given time point) with efficacy in the cohort if at least 2 of 3 patients were responders.
Data exclusions	The data analysis was based on all 12 participants
Replication	Dark adaptation bleaching and recovery in the study eye with short wavelength stimulus of > 1.0 log unit at 2 of 3 measurements obtained prior to treatment.
Randomization	Non-randomized trial. Safety and efficacy trial, all patients were treated in the non-dominant eye (poorer seeing eye), except for one participant who met all inclusion criteria only in the dominant eye.
Blinding	This Phase 1/2 trial was partially masked. The participants were not masked. The treating physicians and personnel at the surgical location (surgeons, anesthesiologist, operating room personnel and others) were not masked. At the clinical site, there were masked ophthalmologists. The remaining assessors at the clinical site (ophthalmologist, study nurse, ophthalmic technician, etc) doing the ophthalmic examinations were masked to the study (treated) eye.

Reporting for specific materials, systems and methods

We require information from authors about some types of materials, experimental systems and methods used in many studies. Here, indicate whether each material, system or method listed is relevant to your study. If you are not sure if a list item applies to your research, read the appropriate section before selecting a response.

Materials & experimental systems

n/a	Involved in the study
☒	☐ Antibodies
☒	☐ Eukaryotic cell lines
☒	☐ Palaeontology and archaeology
☒	☐ Animals and other organisms
☐	☒ Clinical data
☒	☐ Dual use research of concern
☒	☐ Plants

Methods

n/a	Involved in the study
☒	☐ ChIP-seq
☒	☐ Flow cytometry
☒	☐ MRI-based neuroimaging

Clinical data

Policy information about clinical studies

All manuscripts should comply with the ICMJE guidelines for publication of clinical research and a completed CONSORT checklist must be included with all submissions.

Clinical trial registration: Clinicaltrials.gov ref: NCT03374657

Study protocol: The study protocol is available from the corresponding author upon reasonable request

Data collection: All data were collected from the single trial site. Participants were enrolled between Aug 22, 2018 and March 10, 2021. Data was collected and analysed between August 2018 and June 2022.

Outcomes: The primary safety objective was to evaluate the number of participants with adverse events (AEs), serious adverse events (SAEs) and deaths following a single dose of AAV-RLBP1. The primary efficacy objective was to evaluate the kinetics and number of responders in the dark adaptation system after a single dose of AAV-RLBP1. This endpoint was custom-designed and assessed in a 5-year natural history study in patients with RLBP1-RD and was found suitable as a potential primary efficacy endpoint in an exploratory clinical trial for future validation (Burstedt et al 2023). Secondary outcomes included microperimetry, visual field sensitivity, dominant eye test and patient-reported outcomes. There were other objectives that will be analyzed at the end of this study.

Plants

Seed stocks: *Report on the source of all seed stocks or other plant material used. If applicable, state the seed stock centre and catalogue number. If plant specimens were collected from the field, describe the collection location, date and sampling procedures.*

Novel plant genotypes: *Describe the methods by which all novel plant genotypes were produced. This includes those generated by transgenic approaches, gene editing, chemical/radiation-based mutagenesis and hybridization. For transgenic lines, describe the transformation method, the number of independent lines analyzed and the generation upon which experiments were performed. For gene-edited lines, describe the editor used, the endogenous sequence targeted for editing, the targeting guide RNA sequence (if applicable) and how the editor was applied.*

Authentication: *Describe any authentication procedures for each seed stock used or novel genotype generated. Describe any experiments used to assess the effect of a mutation and, where applicable, how potential secondary effects (e.g. second site T-DNA insertions, mosiacism, off-target gene editing) were examined.*
